# FK506-binding protein, FKBP12, promotes serine utilization and negatively regulates threonine deaminase in fission yeast

**DOI:** 10.1016/j.isci.2022.105659

**Published:** 2022-11-24

**Authors:** Mayuki Sasaki, Shinichi Nishimura, Yoko Yashiroda, Akihisa Matsuyama, Hideaki Kakeya, Minoru Yoshida

**Affiliations:** 1 Department of Biotechnology, Graduate School of Agricultural and Life Sciences, The University of Tokyo, Tokyo 113-8657, Japan; 2 Collaborative Research Institute for Innovative Microbiology, The University of Tokyo, Tokyo 113-8657, Japan; 3 Graduate School of Pharmaceutical Sciences, Kyoto University, Kyoto 606-8501, Japan; 4 RIKEN Center for Sustainable Resource Science, Saitama 351-0198, Japan

**Keywords:** Biosynthesis, Biological sciences, Biochemistry, Cell biology

## Abstract

FK506-binding protein with a molecular weight of 12 kDa (FKBP12) is a receptor of the immunosuppressive drugs, FK506 and rapamycin. The physiological functions of FKBP12 remain ambiguous because of its nonessentiality and multifunctionality. Here, we show that FKBP12 promotes the utilization of serine as a nitrogen source and regulates the isoleucine biosynthetic pathway in fission yeast. In screening for small molecules that inhibit serine assimilation, we found that the growth of fission yeast cells in medium supplemented with serine as the sole nitrogen source, but not in glutamate-supplemented medium, was suppressed by FKBP12 inhibitors. Knockout of FKBP12 phenocopied the action of these compounds in serine-supplemented medium. Metabolome analyses and genetic screens identified the threonine deaminase, Tda1, to be regulated downstream of FKBP12. Genetic and biochemical analyses unveiled the negative regulation of Tda1 by FKBP12. Our findings reveal new roles of FKBP12 in amino acid biosynthesis and nitrogen metabolism homeostasis.

## Highlights

• FK506, rapamycin, and SLF suppress serine utilization
• FKBP12 promotes serine utilization
• FKBP12 suppresses threonine deaminase and isoleucine biosynthesis
• Threonine deaminase is regulated by multiple mechanisms

## Graphical abstract

**Figure:**
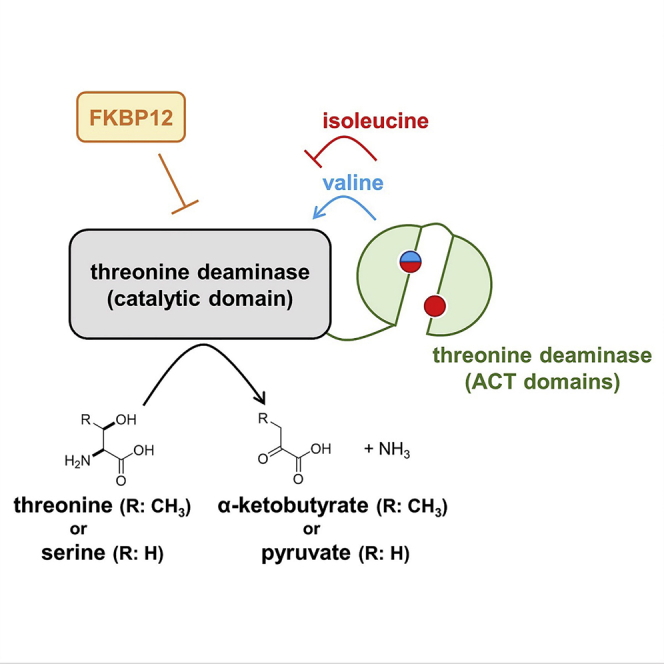

## Introduction

FKBP12 is widely distributed among eukaryotes, from yeast to mammals, and is ubiquitously expressed in cells.^1^^,^^2^ This protein was originally identified in human and bovine tissues as a cellular receptor of FK506, an immunosuppressive drug.^3^^,^^4^ The FKBP12-FK506 complex further binds to calcineurin to inhibit its phosphatase activity, which suppresses the expression of interleukin-2 (IL-2) and the immune response of T cells.^5^^,^^6^^,^^7^ FKBP12 is also a receptor of rapamycin, another immunosuppressant that shows anti-tumor activity.^8^ The FKBP12-rapamycin complex binds to the mechanistic/mammalian target of rapamycin complex-1 (mTORC1), which inhibits mTORC1 activity to halt cell proliferation.^6^ The minimum structure of FK506 and rapamycin that shows potent inhibitory activity against FKBP12 was extracted to design SLF (synthetic ligand of FKBP12).^9^ The atomic structures of the FKBP12-drug complexes and their pharmacological consequences have been extensively studied.^10^^,^^11^ In contrast, the physiological functions of FKBP12 largely remain to be clarified, although it does have peptidyl-prolyl isomerase activity that catalyzes the *cis*-*trans* isomerization of peptidyl-prolyl bonds in client proteins.

Mammalian FKBP12 binds to ryanodine receptors,^2^ inositol-1,4,5 tri-phosphate receptor,^12^ transforming growth factor β/activin-like receptors,^2^ the zinc finger transcription factor, YY1,^13^ and palmitoylated H-Ras.^14^ The diversity of these binding proteins is consistent with the diverse physiological functions of FKBP12 in mammals. FKBP12 is conserved in unicellular eukaryotes, such as the budding yeast, *Saccharomyces cerevisiae*, and is encoded by the *FPR1* gene. Four functions of yeast FKBP12 have been reported: homoserine biosynthesis, ribosomal protein gene expression, regulation of calcineurin activity, and suppression of protein aggregation. FKBP12 regulates aspartate pathway flux by binding to the aspartokinase, Hom3, the first enzyme in the metabolic pathway that generates homoserine from aspartate.^15^^,^^16^ Homoserine is the precursor of the amino acids threonine, methionine, and isoleucine. FKBP12 functions as a transcription factor for ribosomal protein genes cooperatively with Hmo1, a nonhistone chromatin-binding protein.^17^ Simultaneous deletion of FKBP12 and Hmo1 is synthetically lethal, while FKBP12 physically interacts with Hmo1.^18^ Interestingly, FKBP12 physically interacts with calcineurin to regulate its activity.^19^ Recently, budding yeast FKBP12 was shown to suppress protein aggregation *in vitro*, indicating a chaperon function.^20^ In the fission yeast *Schizosaccharomyces pombe*, physiological roles of FKBP12, encoded by the *fkh1* gene, have not been reported, although the effects of rapamycin occur through FKBP12^21^ and FK506 phenocopies mutants lacking the calcineurin catalytic subunit, Ppb1.^22^^,^^23^

Threonine deaminase (TD) catalyzes the deamination of threonine to produce α-ketobutyrate and ammonia, which is the first step in the isoleucine biosynthesis pathway (Figure S1A).^24^^,^^25^^,^^26^ TD is widely distributed from bacteria to fungi and plants but is not present in mammals. TD provided one of the first examples of negative feedback regulation, first reported in 1956.^27^ The pathway end product, isoleucine, inhibits the activity of TD; therefore, the TD reaction is the rate-limiting step in isoleucine biosynthesis. TD consists of an *N*-terminal catalytic domain and two C-terminal regulatory domains, called ACT domains (Figure S1B).^28^ Two molecules of isoleucine bind to the tandem ACT domains, and the binding of the second isoleucine changes the conformation of TD into an inactive state.^29^ Valine is another allosteric regulator of TD, which reverses isoleucine-mediated inhibition of TD by competitively binding to the first isoleucine binding site.^29^^,^^30^ TDs from *Escherichia coli* and the plant *Arabidopsis thaliana* form tetramers, and after binding to isoleucine the plant TD forms an inactive homodimer.^31^^,^^32^ Crystallography analyses suggest that the topology of the regulatory domain is changed by binding to allosteric regulators, thereby shielding the catalytic pockets^32^; however, the structural changes underlying this allosteric regulation are poorly understood.

In this study, we show evidence that the activity of the TD encoded by the *tda1* gene is regulated by FKBP12 in fission yeast. Tda1 is essential for cell growth, and therefore knockdown of *tda1* impaired cell growth. Suppression of cell growth in TD-knockdown cells was rescued by supplementation with isoleucine and by FKBP12 inhibitor treatment or FKBP12 knockout. Furthermore, biochemical analyses revealed that knockout of *fkh1* increased TD activity. These results indicate that FKBP12 suppresses TD to regulate nitrogen metabolism, specifically isoleucine biosynthesis.

## Results

### FKBP12 inhibition suppresses cell growth in serine medium

**Figure 1:**
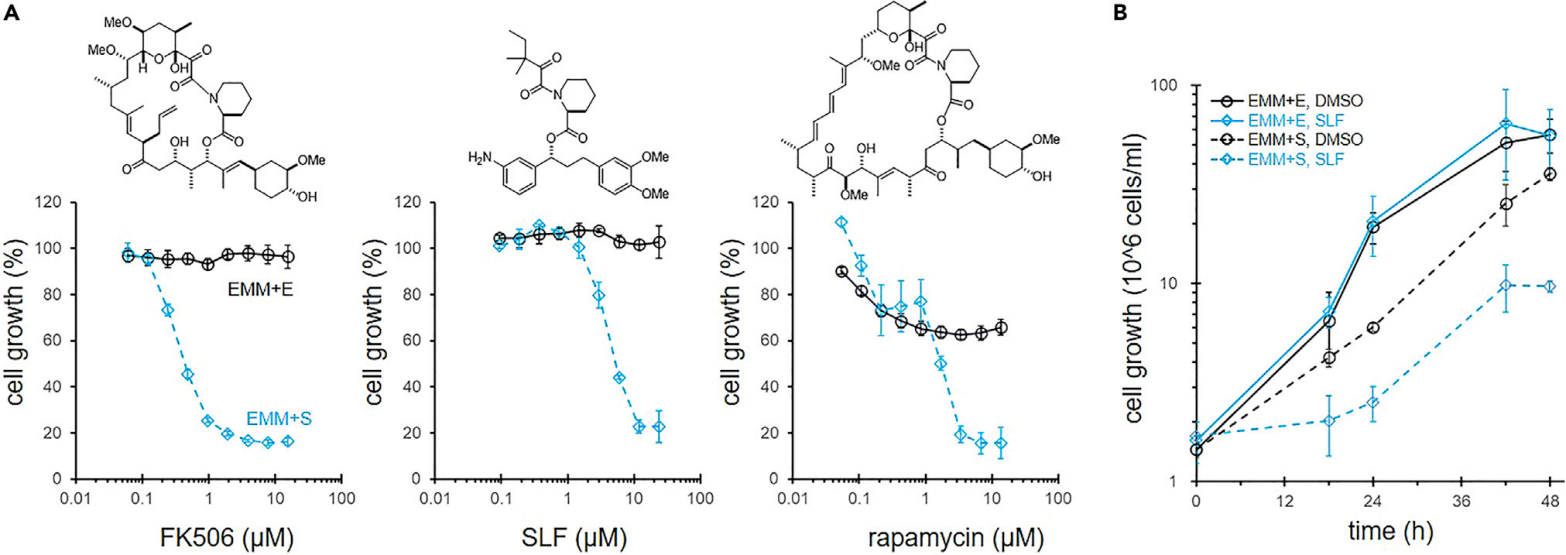
FKBP12i suppresses cell growth in the serine medium (A) Growth inhibition by FKBP12i. Wild-type cells were treated with FK506, SLF, or rapamycin in EMM + E (solid black line) or EMM + S (blue dashed line) medium for 48 h. Data represent the mean ± SE (n = 3). (B) Time-course analyses of the effect of SLF on wild-type cells. Cell numbers were counted in EMM + E or EMM + S medium, in the absence or presence of SLF (12.5 μg/mL, 23.8 μM). Data represent the mean ± SD (n = 3).

The fission yeast *S*. *pombe* can grow when serine is used as a sole nitrogen source (the Edinburgh minimal medium (EMM) + S medium), although its growth is slightly less than that in a medium with a rich nitrogen source, such as glutamate.^33^ We postulated that chemicals that suppress cell growth specifically in EMM + S medium will perturb serine metabolism in cells. To identify such compounds, we conducted screening for compounds that inhibit cell growth of fission yeast cells cultivated in EMM + S medium but not in EMM + E medium containing glutamate as a sole nitrogen source. As a result of screening microbial culture broth collections and an in-house chemical library,^34^^,^^35^ we found that FK506 specifically suppressed growth in EMM + S medium (Figure 1A). The primary target of FK506 in cells is FKBP12; therefore, we tested whether SLF and rapamycin, other FKBP12 inhibitors, can recapitulate the activity of FK506. Both chemicals suppressed cell growth specifically in EMM + S medium, similar to FK506. Rapamycin showed partial growth suppression in EMM + E medium, but the suppression was more drastic in EMM + S medium at higher concentrations (3.4–13.7 μM). We next examined the time-dependence of the SLF effect (Figure 1B). Although SLF did not inhibit growth in EMM + E medium, cell growth in EMM + S medium was almost completely inhibited by SLF for 18 h after addition; slow recovery of cell growth was then observed. These results indicate that inactivation of FKBP12 was responsible for the growth inhibition in EMM + S medium.

### Deletion of the *fkh1* gene suppressed cell growth in serine medium

**Figure 2:**
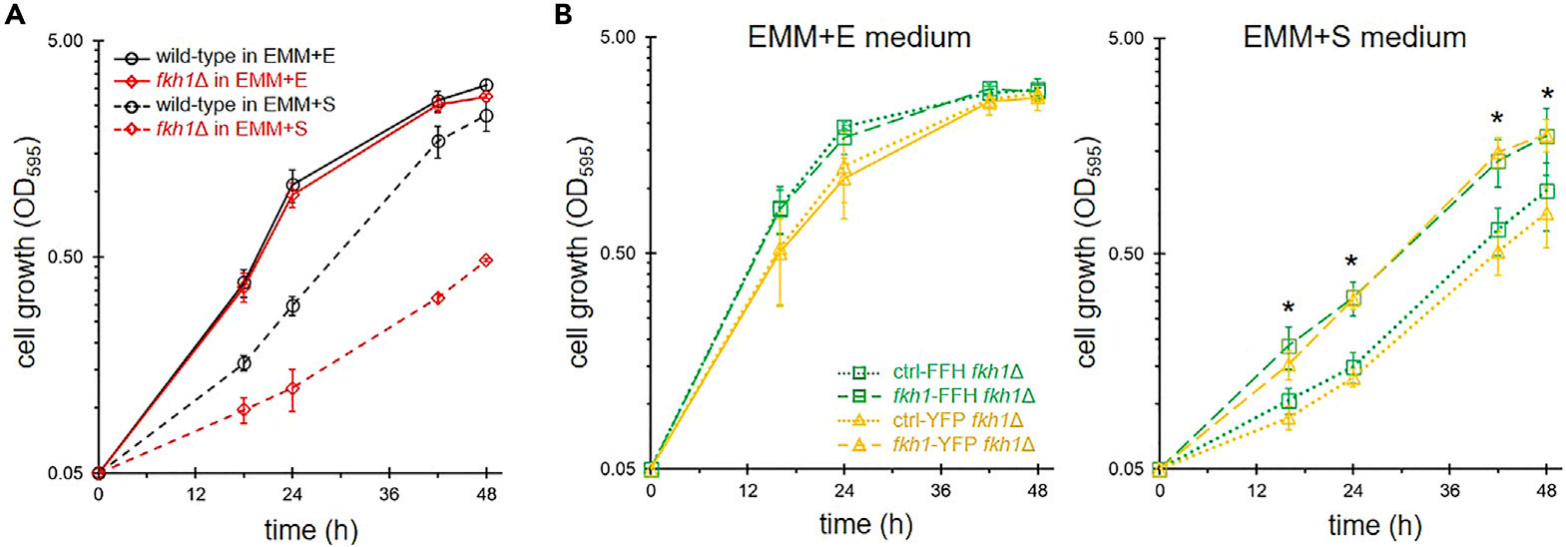
Knockout of FKBP12 showed attenuated growth in the serine medium (A) Growth of wild-type and *fkh1*Δ cells. Cells were cultivated in EMM + E (solid line) or EMM + S (dashed line) medium. Data represent the mean ± SD (n = 3). (B) Recovery of cell growth by *fkh1* expression. *C*-terminally tagged *fkh1* sequences (*fkh1*-FFH or *fkh1*-YFH) were integrated in the *leu1-32* chromosomal locus and expressed under the control of the *nmt1* promoter. FFH and YFH denote FLAG_2_-His_6_ and YFP-FLAG-His_6_, respectively.^44^ Data represent the mean ± SD (n = 3). ∗: p < 0.05 for both ctrl-FFH vs *fkh1*-FFH and ctrl-YFP vs *fkh1*-YFP.

FKBP12 is encoded by the *fkh1* gene in *S*. *pombe*. To examine whether a mutation in the *fkh1* gene results in a similar phenotype to that caused by inhibition of FKBP12, we examined the effects of *fkh1* deletion (*fkh1*Δ) on cell growth. In a time course analysis, *fkh1*Δ cells phenocopied the SLF treatment of wild-type cells (Figures 1B and 2A). *fkh1*Δ cells showed cell growth comparable to that of wild-type cells in EMM + E medium, while in serine medium, they showed a decrease in cell growth. The effect of gene deletion was complemented by expressing C-terminally tagged Fkh1 (Figure 2B). Cells expressing FFH (FLAG_2_-His_6_)-tagged or yellow fluorescent protein (YFP)-tagged Fkh1 grew better than cells expressing only tag sequences in the EMM + S medium. All these data unambiguously show that FKBP12 is required for normal cell growth under serine medium conditions.

### Effect of SLF on cell metabolism

**Figure 3:**
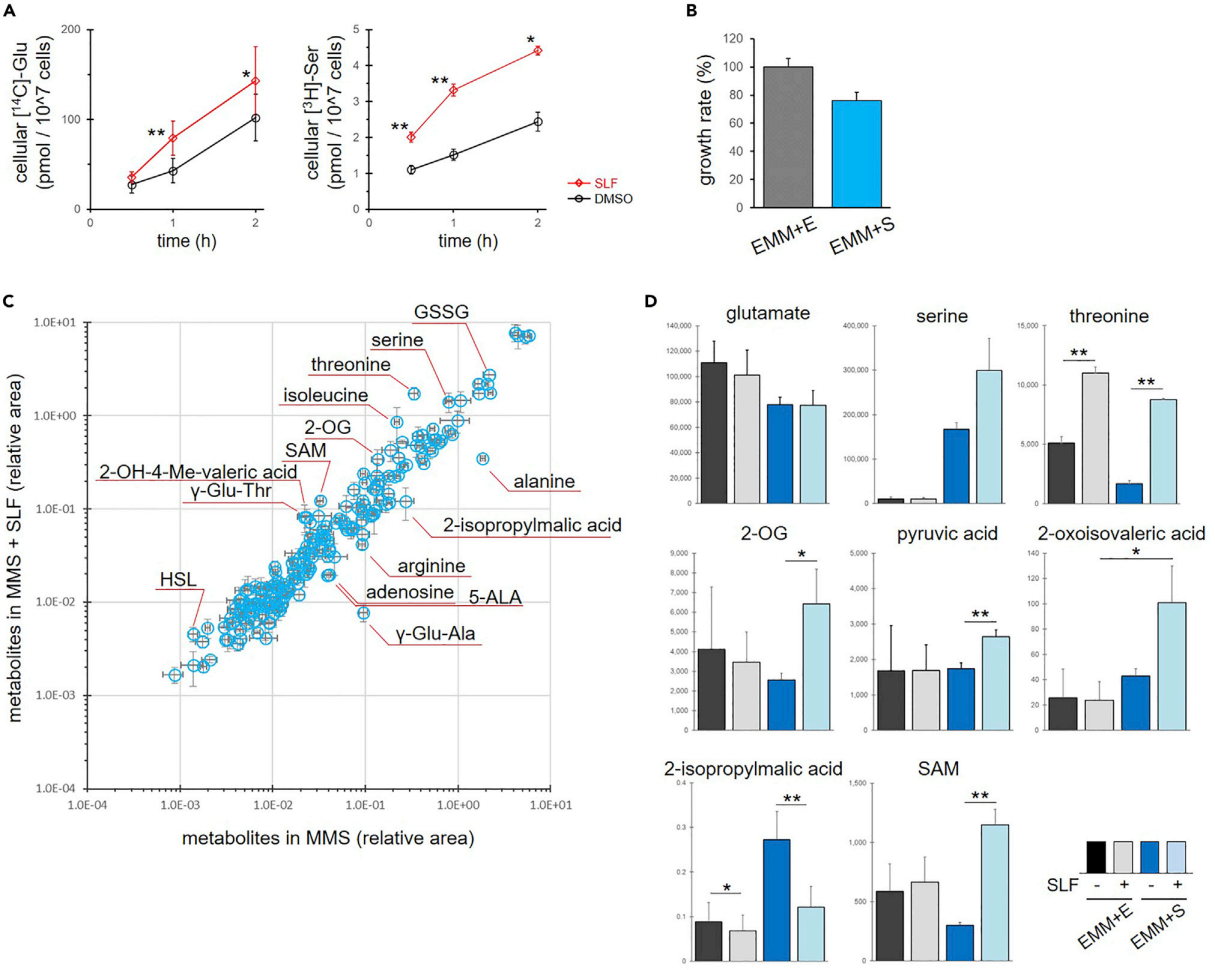
Effect of SLF on amino acid uptake and cellular metabolism (A) Effect of SLF on amino acid uptake. Cellular uptake of RI-labeled amino acids, [^14^C]-glutamate (left) or [^3^H]-serine (right), was measured in the absence (black) or presence (red) of SLF (23.8 μM). Data represent the mean ± SE (n = 5). ∗p < 0.05, ∗∗p < 0.01. (B) Effect of SLF on cell growth. Growth rate, the ratio of the cell number (cells w/SLF ver. cells w/o SLF) are shown. Cells were treated with SLF (23.8 μM) for 6 h, and cell number was counted. These cells were subjected to metabolome analyses. Data represent the mean ± SD (n = 3). (C) Dot plot of metabolites detected in cells cultivated in EMM + S medium. Data represent the mean ± SD (n = 3). (D) Metabolite levels of cells cultivated in EMM + E or EMM + S medium, in the presence or absence of SLF. Representative metabolites are shown. y axis shows the relative area of metabolites detected in the CE-TOFMS spectra or the absolute concentration of metabolites (pmol/10ˆ8 cells). Data represent the mean ± SD (n = 3). ∗p < 0.05, ∗∗p < 0.01.

FKBP12 has diverse binding proteins in yeast and humans; however, the involvement of FKBP12 in serine metabolism has not been reported. To understand how FKBP12 is involved in serine metabolism, we first investigated the possibility that SLF selectively suppresses the uptake of serine into cells. Fission yeast cells took up [^14^C]-glutamate or [^3^H]-serine in a time-dependent manner, although the uptake of glutamate was higher than that of serine (Figure 3A). When cells were exposed to SLF, the cellular levels of both [^14^C]-glutamate and [^3^H]-serine were not decreased but instead increased compared with those of DMSO-treated cells. Consistent with this, the growth defect of *fkh1*Δ cells in EMM + S medium (5 mM) was not attenuated by supplementation with a higher concentration of serine (40 mM) (Figure S2). These results ruled out the possibility that SLF inhibits serine uptake and indicates that FKBP12 modulates cell metabolism after serine uptake.

To investigate changes in cell metabolism caused by SLF, we conducted metabolome analyses. Fission yeast cells were cultivated in EMM + E or EMM + S medium and then treated with DMSO or SLF for 6 h (Figure 3B). Cells were harvested, and the hydrophilic metabolites were extracted and subjected to capillary electrophoresis-mass spectrometry. We detected 322 peaks, most of which were annotated (Tables S1 and S2). The amounts of 51 metabolites differed between EMM + E and EMM + S media. The amounts of only 13 metabolites were changed by SLF in EMM + E medium, while amounts of 76 metabolites were changed in EMM + S medium (Figures 3C and S3). Six metabolites, including threonine, were detected as commonly changed by SLF.

When the cellular level of glutamate was compared between cells cultivated in EMM + E and EMM + S media, the former was only slightly higher. In contrast, the cellular level of serine was substantially higher in the latter. This indicated that serine assimilation was less efficient than glutamate assimilation (Figure 3D). The amount of serine in cells grown in EMM + S medium was further increased by SLF, indicating that serine assimilation but not serine uptake was inhibited by SLF, which was consistent with the result of the radioisotope (RI) experiments (Figure 3A). Threonine showed similar behavior to serine. In contrast, nine amino acids, including alanine, were decreased by SLF, indicating that the nitrogen source was starving, although the most abundant amino acid, glutamate, was tolerant to SLF (Figures 3D and S4). In fact, SLF induced a significant increase in 2-oxoglutarate, the precursor of glutamate, which is a general metabolic feature under nitrogen starvation.^36^^,^^37^ SLF increased the levels of other α-ketoacids, pyruvic acid, 2-oxoisovaleric acid, and phenylpyruvic acid, which are the deaminated metabolites of alanine, valine, and phenylalanine, respectively (Figure 3D). SLF also increased levels of the metabolites 4-methyl-2-oxovaleric acid and 3-methyl-2-oxovaleric acid, which are deaminated metabolites of leucine and isoleucine, respectively, and decreased the levels of the reduced metabolite, 2-hydroxy-4-methylvaleric acid (Figure S5).

SLF treatment in EMM + S medium increased the cellular levels of multiple serine-related metabolites, for example, *O*-phosphoserine and *O*-phosphoglyceric acid, which are precursors of serine biosynthesis, and also *N*-acetylserine and *O*-acetylserine, which indicate excess accumulation of serine by SLF (Figure S5 and S6). Serine is the carbon source for the one carbon pathway. *S*-adenosylmethionine (SAM) and other methylated metabolites were increased by SLF in EMM + S medium (Figures 3D and S5), indicating that SLF treatment does not inhibit serine hydroxymethyltransferase.

### Screening for proteins downstream of FKBP12

To identify molecules downstream of FKBP12, we screened mutant cells lacking genes involved in serine metabolism (Figure S6 and Table S3). Gene deletion mutants for nine nonessential genes were prepared, and their growth was tested in glutamate and serine media (Figure S7). Two gene deletion mutants, *shm1*Δ and *fsf1*Δ, showed poor growth in EMM + S medium. *shm1* encodes serine hydroxymethyltransferase, which catalyzes the formation of 5,10-methylenetetrahydrofolate from tetrahydrofolate. The *shm1*Δ cells showed growth defects in both glutamate and serine media, indicating that Shm1 and Fkh1 function in different pathways. In fact, the metabolome analyses implied that the one carbon cycle was not inhibited by SLF. The *fsf1* gene encodes a putative serine transporter, predicted to be localized in the mitochondrial inner membrane. To examine if Fsf1 interacts with FKBP12, we prepared double gene deletion mutants and tested their growth in EMM + S medium (Figure S7C). The *fsf1*Δ cells showed slower growth than wild-type cells in the serine medium. The *fsf1*Δ *fkh1*Δ cells showed more severe growth inhibition, indicating that Fsf1 and Fkh1 function in different pathways. Fkh1 may therefore not be involved in mitochondrial import of serine.

**Figure 4:**
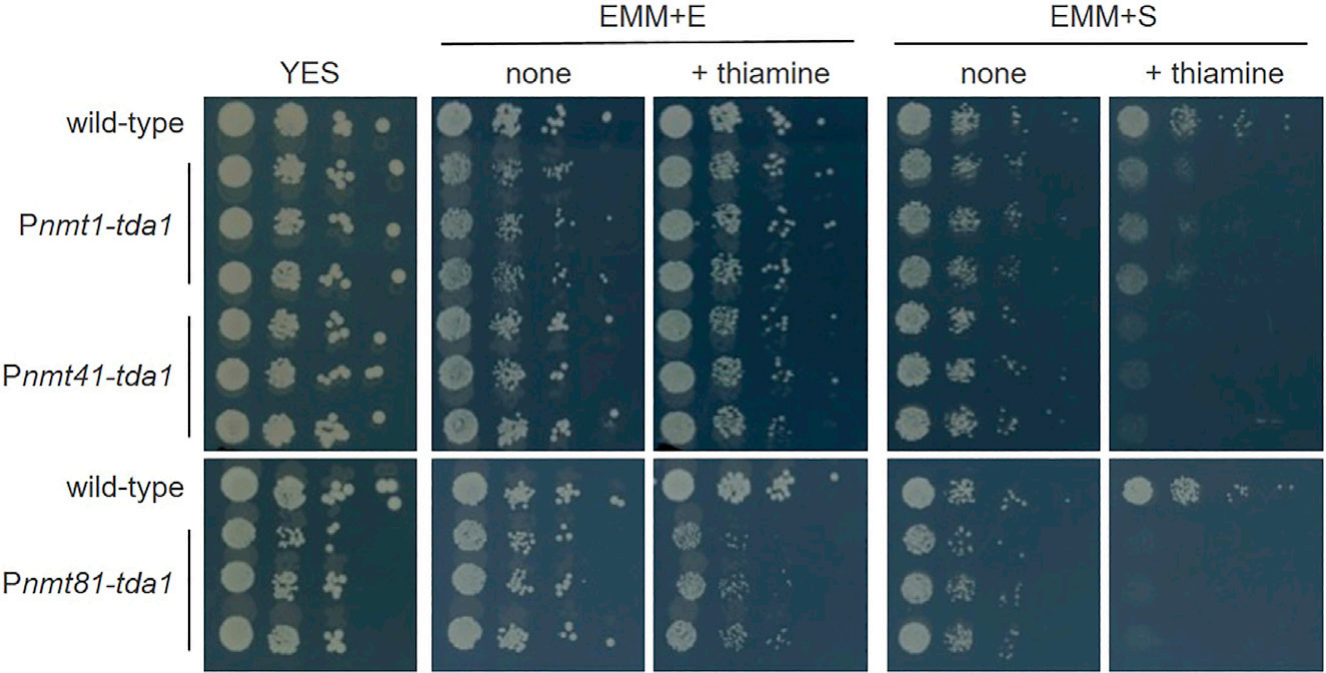
Effect of Tda1 expression level on cell growth Wild-type cells and engineered cells expressing *tda1* under three promoters were inoculated on a rich medium YES, synthetic EMM + E or EMM + S medium for five days. Thiamine (10 μM) was added to suppress the promoter activity. Representative results of three independent experiments are shown.

The experiments using mutant cells lacking nonessential genes did not suggest the downstream protein of FKBP12. We next examined knockdown of an essential gene. Among several essential genes involved in serine metabolism (Figure S6 and Table S3), we focused on Tda1, an enzyme that catalyzes the deamination of threonine.^38^ Metabolome analyses revealed the accumulation of threonine and serine by SLF; therefore, TD was a possible target protein (Figure 3D). Plant homologs of Tda1 also recognize serine as their substrate in spite of a lower affinity than threonine.^39^ To shut off *tda1* expression, we employed an inducible promoter of the *nmt1* gene and its variants: P*nmt1*, P*nmt41*, and P*nmt81*.^40^^,^^41^ We inserted the promoters into the genomic locus of *tda1* and examined the growth of engineered cells in EMM + E and EMM + S medium. In EMM + E medium, substitution of the promoter showed no effect, but addition of thiamine to the medium to shut off *tda1* expression attenuated the growth of P*nmt81*-*tda1* cells (Figure 4). In EMM + S medium, P*nmt41*-*tda1* and P*nmt81*-*tda1* cells showed no growth in the presence of thiamine, while the growth of P*nmt1*-*tda1* cells was partially inhibited. These results indicated that Tda1 is required for cell growth in EMM + E medium, which was consistent with results from a large-scale analysis that annotated the essentiality of this gene.^42^ In addition, the growth of cells in EMM + S medium requires higher levels of Tda1 than growth in EMM + E medium. These results indicate that Tda1 is required to catabolize serine as a nitrogen source.

### Recovery of growth of *tda1* knockdown mutants by inactivation of Fkh1 or supplementation with isoleucine

**Figure 5:**
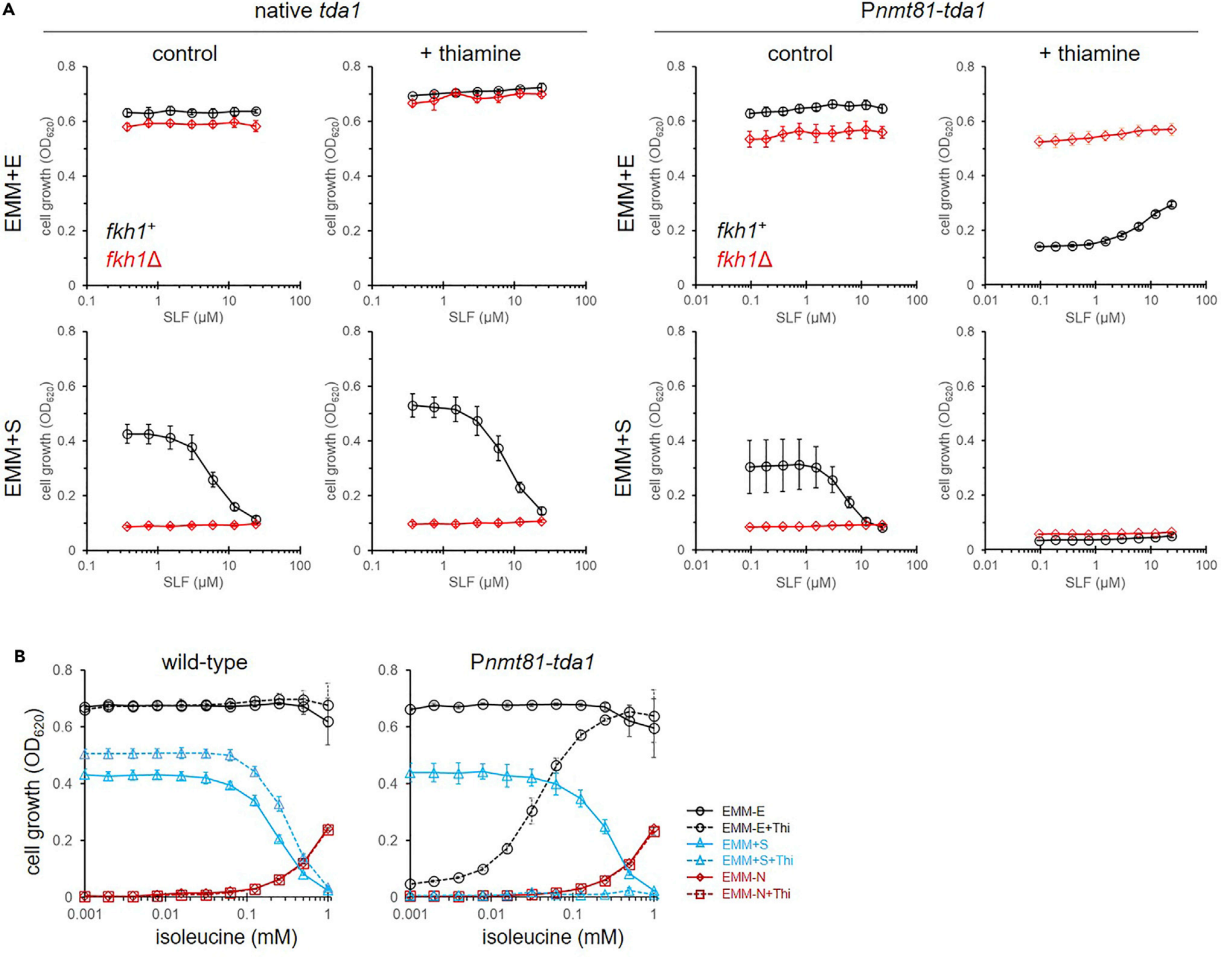
Genetic interactions between *tda1* and *fkh1* (A) Growth and drug sensitivity of wild-type and engineered cells expressing *tda1* under the control of the P*nmt81* promoters. *tda1* expression was modulated in wild-type (black) or *fkh1*Δ (red) cells. Cells were cultivated in EMM + E or EMM + S medium, in the presence of thiamine (10 μM) for 48 h. Data represent the mean ± SE (n = 3). (B) Effect of isoleucine on the growth of cells expressing *tda1* under the control of P*nmt81* promoter. Wild-type or engineered cells were inoculated in EMM + E, EMM + S, or EMM-N medium for 72 h. Thiamine (10 μM) was added to suppress the promoter activity. Data represent the mean ± SE (n = 4).

To investigate the functional relationship between Tda1 and FKBP12, we examined the role of FKBP12 in *tda1*-knockdown cells. In EMM + E medium, P*nmt81*-*tda1* cells showed severe growth inhibition in the presence of thiamine, confirming its essential role in cell growth (Figure 5A). Notably, the deletion of *fkh1* rescued the poor growth of P*nmt81*-*tda1* cells. SLF recovered the growth of *tda1* knockdown cells in a concentration-dependent manner. These results indicate that FKBP12 regulates the function of Tda1 in a negative fashion. The poor growth of P*nmt81*-*tda1* cells in EMM + E medium in the presence of thiamine was recovered by isoleucine, the end product of the Tda1 pathway (Figures 5B and S1). The effective concentration of isoleucine was lower than that for supporting growth in EMM without nitrogen source (EMM-N) medium to which isoleucine was added as the sole nitrogen source. In EMM + S medium, P*nmt81*-*tda1* cells showed no growth in the presence of thiamine, which was not recovered by genetic or pharmacological inactivation of FKBP12 (Figure 5A). Because cells require higher Tda1 activity in EMM + S medium than in EMM + E medium to obtain ammonium from serine (Figure 4), the Tda1 activity in P*nmt81*-*tda1 fkh1*Δ cells might not be sufficient for cell growth. In fact, isoleucine supplementation did not support but inhibited the growth of P*nmt81*-*tda1* cells in EMM + S medium (Figure 5B). This growth inhibition might result from the allosteric inhibition of serine deamination by Tda1 in the presence of isoleucine.

### Downregulation of cellular TD activity by FKBP12

In budding yeast, FKBP12 functions as a transcription factor that regulates the transcription of ribosomal protein genes.^17^ To test the possibility that Fkh1 regulates the gene expression of *tda1*, we examined the transcription level of *tda1* in the presence of SLF. However, *tda1* expression was not changed significantly by SLF in either EMM + E medium or EMM + S medium (Figure S8A). Next, we examined if Fkh1 regulates the subcellular localization of Tda1. Tda1 has a putative mitochondrial targeting signal (Figure S8B), and we detected GFP-fused Tda1 in mitochondria (Figure S8C). However, the localization was not changed in the absence of Fkh1. Curiously, C-terminal tagging of Tda1 inhibited yeast growth in EMM + S medium but not in EMM + E medium (Figure S8D). C-terminal modification may cause partial inhibition of Tda1 activity.

**Figure 6:**
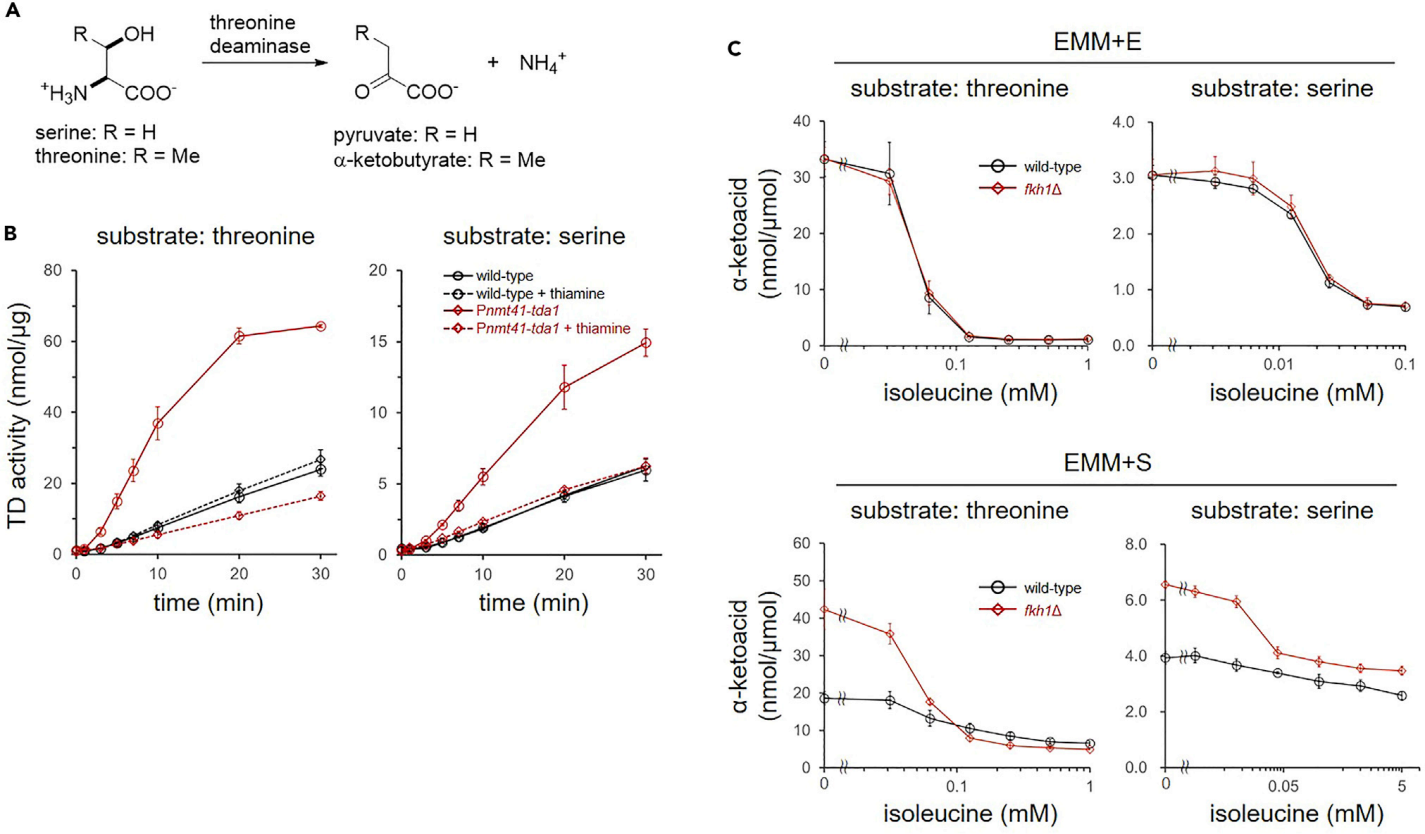
Effect of *fkh1* deletion on TD activity (A) Deamination of threonine and serine by Tda1. The amount of the produced α-ketoacids was quantified in the TD assay. (B) TD activity of cell lysates. Cell lysates from wild-type or engineered cells expressing *tda1* under the control of P*nmt41* promoter were used in the enzymatic assay. Cells were cultivated in EMM + S medium in the presence or absence of thiamine (10 μM). Threonine (5 mM) or serine (20 mM) was used as a substrate. Data represent the mean ± SD (n = 3). (C) Cell lysates of wild-type or *fkh1*Δ cells were used to examine the cellular TD activity in the presence of isoleucine. Threonine (10 mM) or serine (20 mM) was used as a substrate. Reaction mixtures were incubated for 10 min. Data represent the mean ± SD (n = 3).

Next, the cellular activity of Tda1 was examined. Using a previously reported method,^38^ we successfully detected TD activity in crude cell lysate (Figures 6A and 6B). Serine was also recognized as its substrate, although the enzymatic activity was lower than that for threonine (Figure 6B). Forced expression of *tda1* by the P*nmt41* promoter induced a substantial increase in enzymatic activity, which was suppressed when its expression was inhibited by thiamine. This result confirmed that Tda1 is responsible for the cellular threonine/serine deaminase activity in fission yeast. The difference in TD activity between wild-type and *fkh1*Δ cell lysates was negligible when cells were cultivated in EMM + E medium (Figure 6C). In contrast, the TD activity of the *fkh1*Δ cell lysate was higher than that of the wild-type cells when cells were cultivated in EMM + S medium. These activities did not depend on whether the substrate was threonine or serine.

Tda1 is allosterically regulated by isoleucine, the end product of the Tda1 pathway. The plant homolog of Tda1 has two binding sites for isoleucine in the C-terminal ACT domains.^29^ To investigate if the allosteric regulation is affected by FKBP12, we tested the effect of isoleucine on the TD activity of wild-type and *fkh1*Δ cells (Figure 6C). When cells were cultivated in EMM + E medium, isoleucine suppressed TD activity in a concentration-dependent manner, independent of the presence of FKBP12. When wild-type cells were cultivated in EMM + S medium, TD activity was moderately suppressed by isoleucine. However, TD activity in the *fkh1*Δ cell lysate was significantly sensitized to isoleucine. This result indicates that FKBP12 downregulates TD activity in the EMM + S medium, probably by changing the conformation of the ACT domains. It is notable that FKBP12 inhibition had little effect on cellular TD activity, indicating that FKBP12 acts on Tda1 in an irreversible manner (Figure S9).

## Discussion

In this study, we identified the fission yeast TD, Tda1, to be downstream of FKBP12. TD catalyzes the deamination of threonine to produce α-ketobutyrate and an ammonium ion. α-ketobutyrate is then further metabolized to synthesize isoleucine. Tda1 is one of the first enzymes for which feedback regulation was described. The activity of Tda1 is inhibited by isoleucine in many organisms. We demonstrated that cell growth in EMM + E medium was suppressed by minimizing the expression of Tda1, which was recovered by inactivating FKBP12 or supplementation with isoleucine, indicating that the fission yeast Tda1 is negatively regulated also by FKBP12.

**Figure 7:**
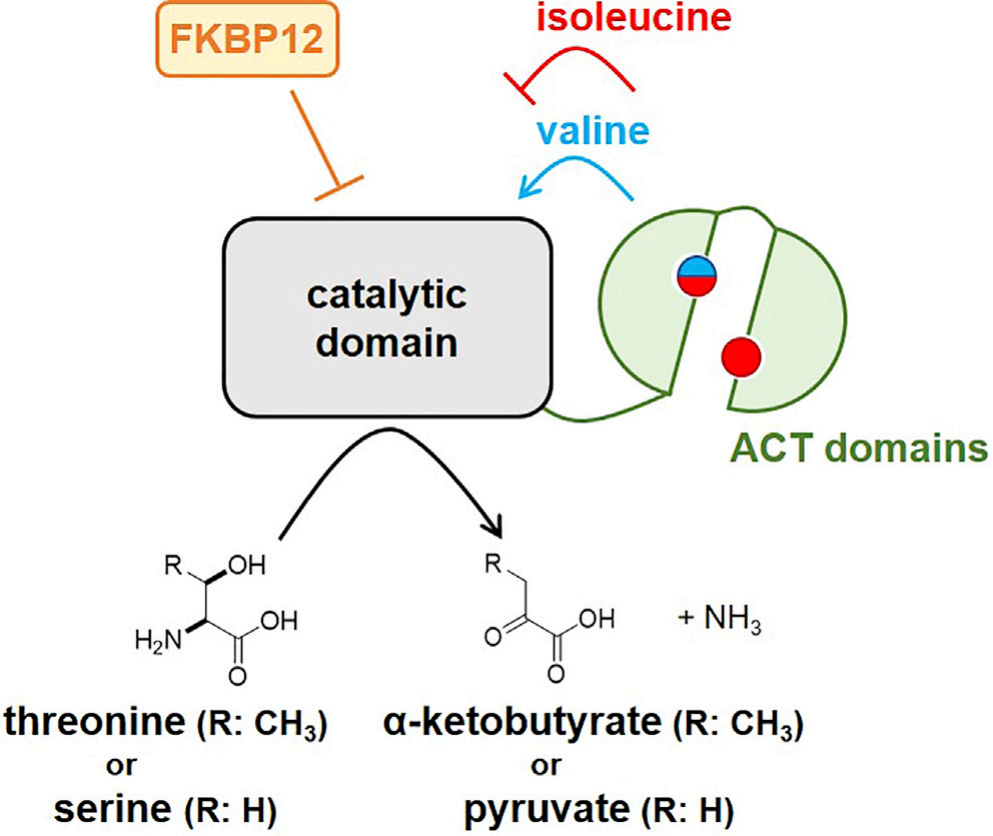
Plausible model for regulation of Tda1 activity The activity of Tda1 is regulated by amino acids, isoleucine (red), and valine (blue), which bind to the regulatory ACT domains. Valine binds to one of the isoleucine binding sites. In this study, FKBP12 was shown to downregulate the activity of Tda1, probably through regulating the structure of ACT domains.

TD consists of a catalytic domain and two C-terminal ACT domains (Figure S1). Fission yeast Tda1 is a mitochondrial protein that has a putative mitochondrial targeting sequence on the N-terminal side of the catalytic domain (Figure S8). The catalytic lysine residue for tethering the cofactor pyridoxal phosphate (PLP) and PLP-binding motifs for binding to the phosphate group of PLP are conserved in fission yeast Tda1 (Figure S1). Isoleucine and valine binding sites are also conserved in the two ACT domains. Fission yeast TD activity was previously shown to be inhibited by isoleucine.^38^ We also observed that isoleucine inhibited Tda1 activity when cells were cultivated in EMM + E medium (Figure 6). In contrast, Tda1 activity of cells cultivated in EMM + S medium was low and only moderately inhibited by isoleucine. Tda1 activity in EMM + S medium was increased and sensitized to isoleucine when *fkh1* was knocked out (Figure 6). These results indicate that the conformation of Tda1 in EMM + S medium is different from that in EMM + E medium, probably due to the change in the structure of the ACT domains, which could be regulated by Fkh1 (Figure 7). A limitation of this study is that the enzyme activity was only examined in crude cell lysates, which contain amino acids such as isoleucine and valine. Furthermore, we could not evaluate the endogenous protein level of Tda1 because tagging was not successful. To demonstrate the above hypothesis, *in vitro* experiments such as kinetic and structural analyses using amino acid-free, purified proteins are required.

The aspartokinase, Hom3, is an FKBP12-binding protein in the budding yeast *S*. *cerevisiae*. Hom3 catalyzes the phosphorylation of aspartate, which is the first step in threonine biosynthesis. Genetic analyses indicate that FKBP12 is required for feedback inhibition of Hom3 by threonine.^16^ The physical interaction of FKBP12 and Hom3 was enhanced by threonine, although changes in aspartokinase activity were not demonstrated by the addition of FKBP12. The physical interaction between the fission yeast aspartokinase, SPBC19F5.04, and FKBP12 was also detected in a proteome-wide analysis.^43^ It is therefore possible that the increase in cellular threonine by SLF treatment (Figures 3 and S3B) is due to the impaired feedback inhibition of SPBC19F5.04 by inactivation of FKBP12. Importantly, both aspartokinase and TD have ACT domains that possess amino acid-binding sites for the allosteric feedback regulation. Our findings suggest that the ACT domain is responsible for interaction with FKBP12.

Tda1 is essential for cell growth because it is responsible for isoleucine biosynthesis. When the expression of *tda1* is suppressed in EMM + E medium, cells cannot grow, but growth can be rescued by supplementation with isoleucine (Figure 5). In contrast, when serine is used as a sole nitrogen source, Tda1 has at least two functions: isoleucine biosynthesis and extracting ammonium from serine. Therefore, the knockdown phenotype of *tda1* was not rescued by isoleucine supplementation. This study started with the finding that FKBP12 inhibitors suppress cell growth in EMM + S medium. However, the molecular mechanism by which FKBP12 inactivation retards growth is ambiguous. When cells were cultivated in EMM + S medium, the cell lysate of *fkh1*Δ cells showed higher TD activity than wild-type cells (Figure 5), while metabolome analyses indicated that SLF treatment suppressed TD activity to cause accumulation of threonine and nitrogen starvation (Figure 3D). One possible explanation for this contradiction is that TD activity in *fkh1*Δ cells cultured in EMM + S medium is more sensitive to isoleucine than that in wild-type cells (Figure 6C), which could impair serine deamination in the presence of isoleucine generated from threonine. Alternatively, the accumulation of pyruvate caused by serine deamination by Tda1 could be a cause of growth inhibition by FKBP12 inactivation, although the molecular mechanism for this is not known.

The negative feedback regulation of Tda1 by isoleucine has been studied since the 1950s. FKBP12 is another new factor that can attenuate Tda1 activity. Molecular- and atomic-level investigations of the interactions between Tda1 and FKBP12 will clarify novel mechanisms of regulation of metabolic enzymes. This will inform the development of new antifungal drugs because humans do not possess TD.

### Limitations of the study

We have demonstrated that TD is negatively regulated by FKBP12 in fission yeast. Whether this interaction is conserved in other organisms is of interest. Furthermore, the molecular mechanisms of the interaction between TD and FKBP12 should be clarified. Finally, the mechanisms underlying the growth suppression by FKBP12 inhibition in serine-supplemented medium is unknown.

## STAR★Methods

### Key resources table

**Table:** 

REAGENT or RESOURCE	SOURCE	IDENTIFIER
**Chemicals, peptides, and recombinant proteins**		

SLF	Cayman	Cat#10007974
FK506	FUJIFILM	Cat#063–06071
Rapamycin	AG Scientific	Cat#R-1018
[^14^C]-glutamate	Perkin Elmer	Cat#NEC290E
[^3^H]-serine	Perkin Elmer	Cat#NET248
ISOGEN	Nippon Gene	Cat#319–90211
MitoTracker Red CMXRos	Thermo Fisher	M7512
pyridoxal phosphate	Fiji film	Cat#165–20943
dinitrophenylhydrazine	Nacalai tesque	Cat#13521–64

**Critical commercial assays**		

PrimeScript RT reagent kit	Takara	RR037A
THUNDERBIRD Next SYBR	TOYOBO	QPS-201

**Experimental models: Organisms/strains**		

NI1145 (*h*^−^*fkh1*::*natR*)	NBRP	FY25848
The *Schizosaccharomyces pombe* strains generated in this manuscript are listed in Table S4.	Shinichi Nishimura	This manuscript

**Oligonucleotides**		

The primers used in this manuscript are listed in Table S5.	Shinichi Nishimura	This manuscript

**Recombinant DNA**		

pFA6a-kanMX6	Jürg Bähler	Bähler et al., 1998
pFA6a-kanMX6-P3nmt1	Jürg Bähler	Bähler et al., 1998
pFA6a-kanMX6-P41nmt1	Jürg Bähler	Bähler et al., 1998
pFA6a-kanMX6-P81nmt1	Jürg Bähler	Bähler et al., 1998
pFA6a-GFP(S65T)-kanMX6	Jürg Bähler	Bähler et al., 1998

### Resource availability

#### Lead contact

Further information and requests for resources and reagents should be directed to and will be fulfilled by the lead contact, Shinichi Nishimura (anshin@g.ecc.u-tokyo.ac.jp).

#### Materials availability

All materials generated in this study are available on request to the lead contact.

### Experimental model and subject details

#### Yeast strains and cultivation

Yeast strains and oligo DNAs used in this study are listed in Tables S4 and S5. Gene deletion mutants without auxotroph markers were generated by random sporulation using the auxotroph Bioneer library v5.0^42^ and wild-type cells, or by a PCR-based strategy, replacing the entire coding region with *kanMX6* module in pFA6a-kanMX6.^45^^,^^46^ Correct gene deletion was confirmed by colony PCR. *C*-terminally tagged Fkh1 was expressed as reported previously.^44^ The forced expression or the shut-off of the expression of *tda1* was carried out as described previously using plasmids pFA6a-kanMX6-P3nmt1, pFA6a-kanMX6-P41nmt1, or pFA6a-kanMX6-P81nmt1.^46^ *C*-terminal tagging by GFP was done using pFA6a-GFP(S65T)-kanMX6.^46^ Yeast cells were cultivated in Edinburgh minimal medium 2 (EMM) without NH_4_Cl,^47^ supplemented with glutamate (2 mM; EMM + E) or serine (2 mM; EMM + S). EMM-N denotes EMM without any nitrogen source. YES is a rich medium composed of yeast extract, glucose, and 225 mg/L each of five supplements (Ade, Ura, l-Leu, l-His, and l-Lys).^47^

### Method details

#### Cell growth test

Cells were cultivated in EMM + E medium overnight and harvested by centrifugation (2,500 rpm, 3 min). Cells were washed by EMM-N medium three times and resuspended in the same medium. Cells (0.3 OD_595_) were cultivated for 24 h at 27°C. Then, cells were inoculated in EMM + E or EMM + S medium with or without nutrients or chemicals. The initial concentration of cells was 0.05 OD_595_ for assays in test tubes or 0.0033 OD_595_ for assays in 96 well plates. Cell growth in 96 well plates was assessed by measuring the turbidity at 595 nm using MULTISKAN FC (Thermo). SLF, FK506, and rapamycin were from Cayman, FUJIFILM, and AG Scientific, respectively. When cells were spotted on solid media, 5- or 10- fold serial dilution series were prepared in EMM-N medium, 5 μL of which were spotted. The highest concentration was 5 × 10ˆ5 cells/mL or 0.015 OD_595_.

#### Uptake of RI-labeled amino acids

Cells were precultured overnight in EMM + E or EMM + S medium. 2 × 10ˆ6 cells were suspended in 400 μL of EMM + E or EMM + S medium. 100 μL of medium containing 2 μCi of [^14^C]-glutamate or [^3^H]-serine was added to the cell suspension, incubated at 27°C for 0.5, 1, or 2 h. Reaction was quenched by ice-cold medium containing 10 mM glutamate or serine (500 μL). Cells were washed by 500 μL of ice-cold medium three times and suspended in 0.5% SDS (100 μL), which was mixed with Scintillation Cocktail (2 mL, Ultima Gold from Perkin Elmer). The RI count was measured using Tri-Carb 4810TR (Perkin Elmer). l-[^14^C(U)]-glutamic acid (281.0 mCi/mmol, 50 μCi/0.5 mL) and l-[^3^H(G)]-serine (30.5 mCi/mmol, 250 μCi/0.25 mL) were purchased from Perkin Elmer.

#### Metabolome analysis

Yeast wild-type cells were treated with SLF (12.5 μg/mL) for 6 h at 27°C. Cells were collected on 0.4 μm Isopore membrane filter (Millipore), after which the trapped cells were washed twice with 10 mL of RO water. The membrane was then immersed in 1,600 μL of methanol in a petri dish with the attached cells facing downwards, followed by ultrasonication for 30 s to dislodge the cells from the filter. The cell extract was then treated with 1,100 μL of Milli-Q water containing internal standards (H3304-1002, Human Metabolome Technologies, Inc. (HMT), Tsuruoka, Yamagata, Japan) and left at room temperature for 30 sec. 2000 μL of the mixture was collected and centrifuged at 2,300 × *g*, 4°C for 5 min. Subsequently, 350 μL of the supernatant was centrifugally filtered through a Millipore 5-kDa cutoff filter (UltrafreeMC-PLHCC, HMT) at 9,100 ×*g*, 4°C for 150 min to remove macromolecules. The filtrate was evaporated to dryness under vacuum and reconstituted in 50 μL of Milli-Q water for metabolome analyses.

Metabolome analysis was conducted according to HMT’s Basic Scan package, using capillary electrophoresis time-of-flight mass spectrometry (CE-TOFMS) based on the methods described previously.^48^^,^^49^ Briefly, CE-TOFMS analysis was carried out using an Agilent CE capillary electrophoresis system equipped with an Agilent 6210 time-of-flight mass spectrometer (Agilent Technologies, Inc., Santa Clara, CA, USA). The systems were controlled by Agilent G2201AA ChemStation software version B.03.01 (Agilent Technologies) and connected by a fused silica capillary (50 μm i.d.×80 cm total length) with commercial electrophoresis buffer (H3301-1001 and I3302-1023 for cation and anion analyses, respectively, HMT) as the electrolyte. The spectrometer was scanned from m/z 50 to 1,000 and peaks were extracted using MasterHands, automatic integration software (Keio University, Tsuruoka, Yamagata, Japan) to obtain peak information including m/z, peak area, and migration time (MT).^50^ Signal peaks corresponding to isotopomers, adduct ions, and other product ions of known metabolites were excluded, and the remaining peaks were annotated according to HMT’s metabolite database based on their m/z values and MTs. Areas of the annotated peaks were then normalized to internal standards and sample amount to obtain relative levels of each metabolite (Table S1). Primary 110 metabolites were absolutely quantified based on one-point calibrations using their respective standard compounds (Table S2).

#### Real-time quantitative PCR

Total RNA was extracted from wild-type cells cultured in EMM + E or EMM + S medium in the presence or absence of SLF (3 mL culture). Cells were harvested by centrifugation, washed by ice-cold water, and suspended in 0.4 mL of ISOGEN (Nippon Gene), to which 0.3 g of glass beads were added. Cells were disrupted with Multi-Beads shocker (Yasui Kikai, 2000 rpm, 10 s). Total RNA was extracted according to the manufacturer’s protocol. The RNA concentration was quantified by a DU730 UV/vis spectrophotometer (Beckman Coulter). cDNA was prepared using the PrimeScript RT reagent kit (TaKaRa). qPCR was performed on Analytikjena qTOWER3G. THUNDERBIRD Next SYBR (TOYOBO) was used for RT-PCR. Primer sequences are listed in Table S5. The expression of *tda1* was normalized to that of *act1*.

#### Microscopy

Cells were cultivated at 27°C. Mitochondria were visualized by MitoTracker Red CMXRos (Thermo Fisher). Yeast cells were incubated with fluorescent dye (50 nM) for 20 min. BZ-X viewer software was used for image acquisition together with KEYENCE BZ-X710 fluorescence microscope equipped with a PlanApo 100x lens.

#### TD enzyme assay

Cell lysate was prepared as described previously.^38^ The cell lysate was finally centrifuged at 15,000 rpm for 10 min at 4°C. The enzymatic assay was conducted as reported previously with some modifications.^51^ Briefly, cell lysate (1 μg for threonine as a substrate or 7 μg for serine as a substrate) in 90 μL of 0.1 M potassium phosphate buffer was mixed with pyridoxal phosphate (PLP) (5 μL of 400 μM) and a variety of concentrations of substrate (threonine or serine) (20 μL), which was incubated at 30°C. The reaction was quenched by 30% TCA (10 μL). 0.2% dinitrophenylhydrazine in 2N HCl (10 μL) was added to the reaction mixture, which was incubated at 30°C for 10 min. Then, 2.5 N NaOH (100 μL) was added to the mixture. After 10 min incubation at room temperature, OD_540_ was measured by MULTISKAN FC (Thermo).

### Quantification and statistical analysis

Statistical analyses were performed as summarized in each figure by using a t-test for comparisons of two groups.

## Data Availability

• Data reported in this paper will be shared by the lead contact on request. • This article does not report original code. • Any additional information required to reanalyze the data reported in this article is available from the lead contact on request.

## Supplemental information

Document S1. Figures S1–S9 and Tables S3–S5

Table S1. List of 322 metabolites with their relative abundance, related to Figure 3

Table S2. List of 110 metabolites with their absolute quantity, related to Figure 3
