# Supplementary material for: FK506-binding protein, FKBP12, promotes serine utilization and negatively regulates threonine deaminase in fission yeast
Source: iScience. 2022 Nov 24;25(12):105659. doi: 10.1016/j.isci.2022.105659 (PMC9730122; doi:10.1016/j.isci.2022.105659)
Supplement: Document S1. Figures S1–S9 and Tables S3–S5 [file mmc1.pdf]

iScience, Volume *25*

Supplemental information

FK506-binding protein, FKBP12, promotes serine utilization and negatively regulates threonine deaminase in fission yeast

Mayuki Sasaki, Shinichi Nishimura, Yoko Yashiroda, Akihisa Matsuyama, Hideaki Kakeya, and Minoru Yoshida

Supplementary materials

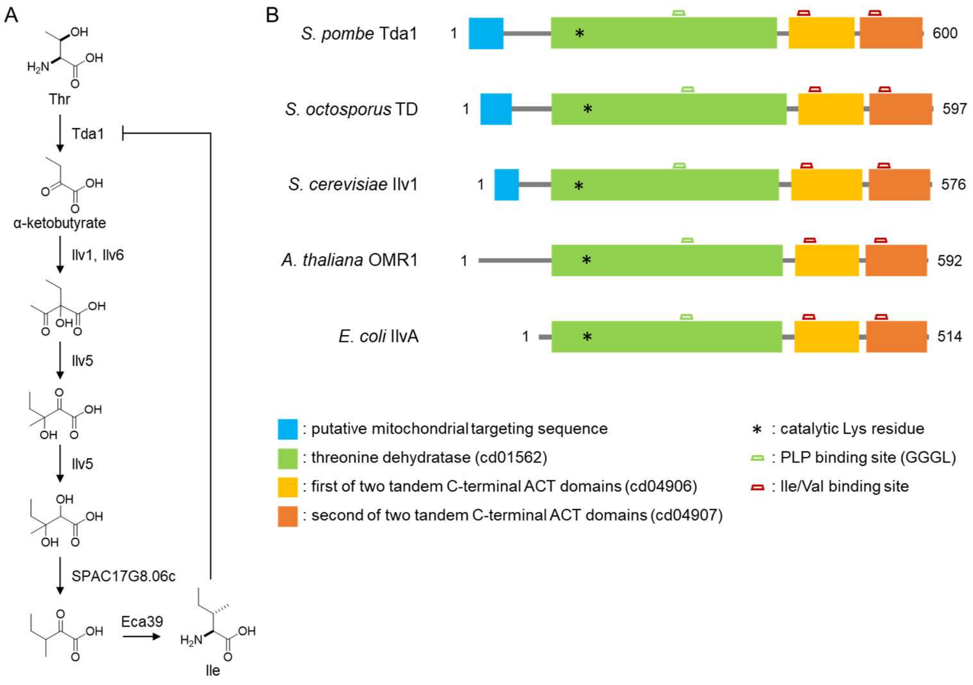

Figure S1. Threonine deaminase in Ile biosynthesis and the domain architectures, related to Figures 6 and 7.

A. Ile biosynthetic pathway. Protein names of the fission yeast homologs are shown.

B. Domain architectures of threonine deaminases. *S. pombe* Tda1 and homologs in *Schizosaccharomyces octosporus* (XP_013019837.1), *S. cerevisiae* (ILV1/YER086W), *Arabodopsis thaliana* (OMR1/NP_187616.1), and *Escherichia coli* (IlvA/P04968) were analyzed by InterPro (https://www.ebi.ac.uk/interpro/).

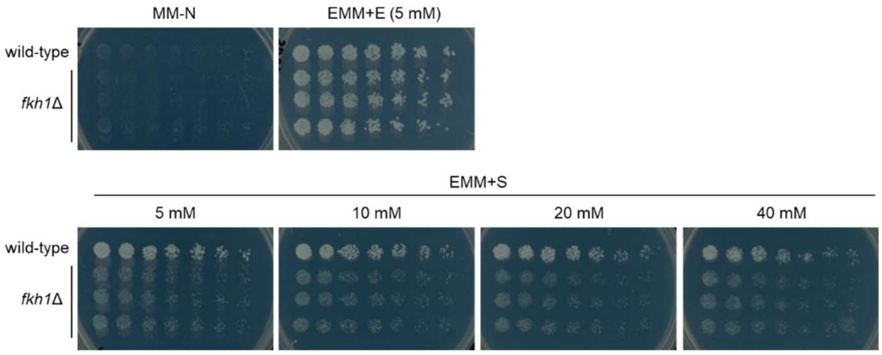

Figure S2. Effect of the concentration of serine on the growth of wild-type and *fkh1*Δ cells, related to Figure 3. Cells were inoculated on the indicated media for 5 days. Representative images of three independent experiments are shown.

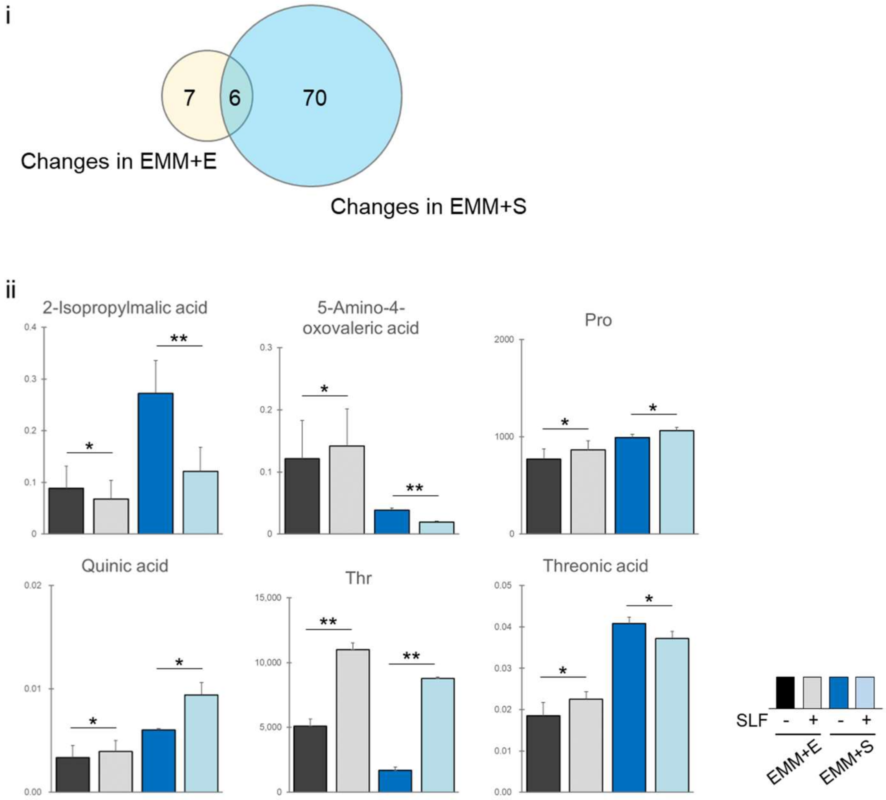

Figure S3. Changes in metabolite levels by SLF, related to Figure 3.

i. Number of metabolites whose cellular levels were changed by SLF with statistical significance are shown. The cellular level of 13 metabolites were affected by SLF in EMM+E medium, while 76 in EMM+S medium. Six metabolites were modulated in both conditions.

ii. Metabolite level of the six common metabolites shown in i. y axis show the relative or absolute amount of the metabolites. Data represent the mean ± SD ($n = 3$). *$p < 0.05$, **$p < 0.01$.

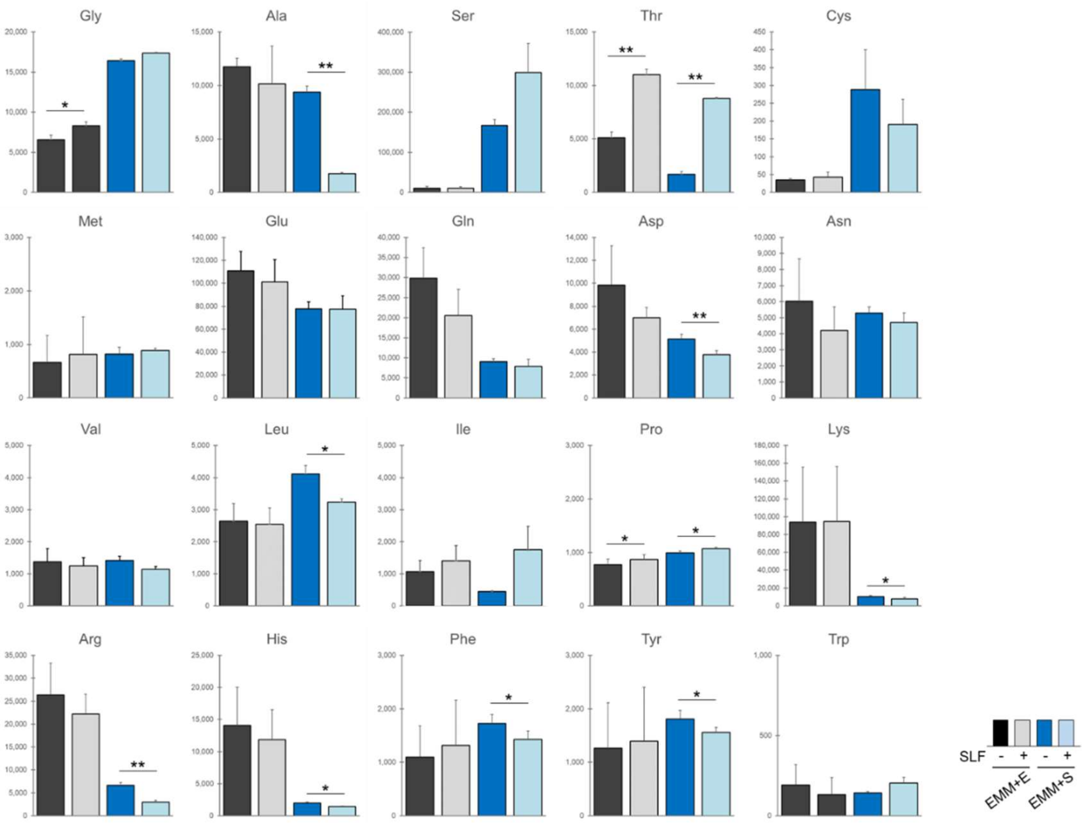

Figure S4. Cellular levels of 20 amino acids, related to Figure 3.

Ile showed an increase in EMM+S medium, despite the lack of statistical significance. Data represent the mean ± SD (n = 3). *$p < 0.05$, **$p < 0.01$.

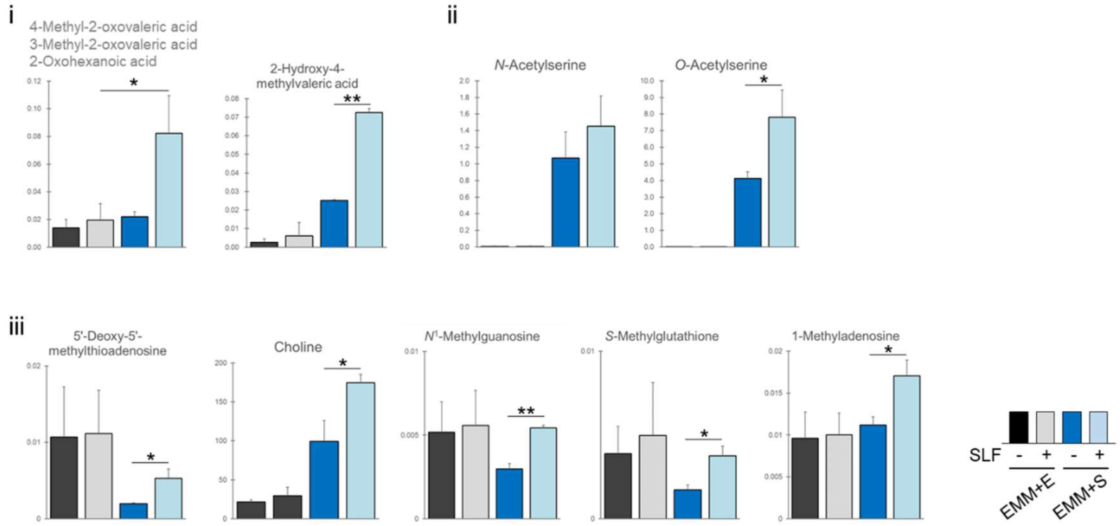

Figure S5, related to Figure 3. Cellular levels of representative metabolites for α-ketoacids (i), serine derivatives (ii), and methylated metabolites (iii). Data represent the mean ± SD (n = 3). *p < 0.05, **p < 0.01.

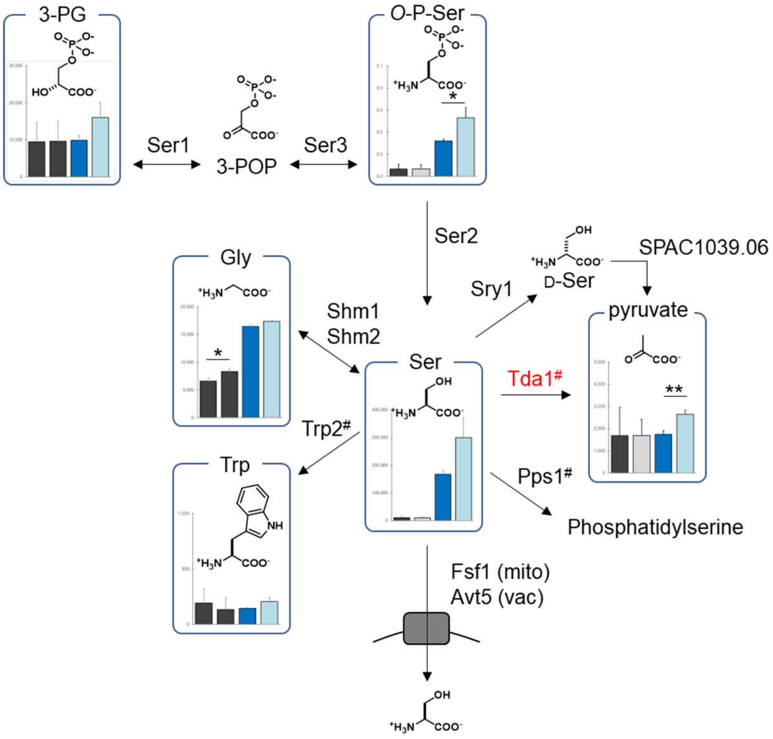

Figure S6, related to Figure 3. Cellular levels of metabolites in serine biosynthesis and assimilation pathway. Data represent the mean ± SD (n = 3). *p < 0.05, **p < 0.01. #Enzymes encoded by essential genes.

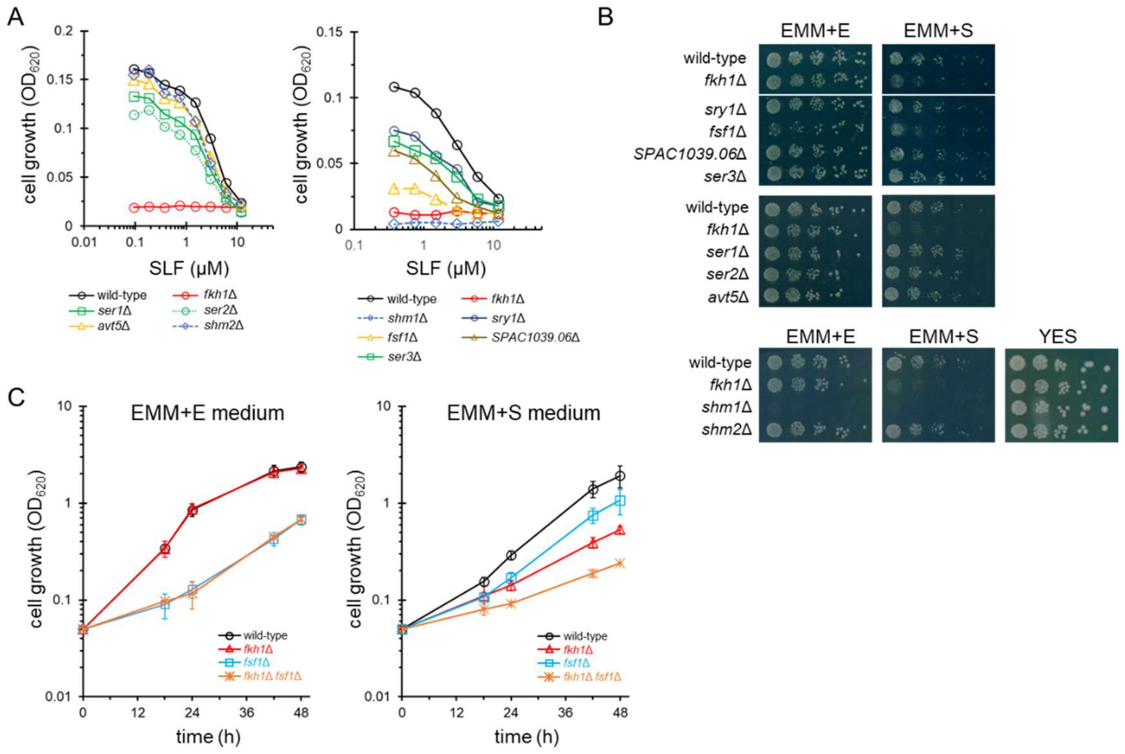

Figure S7. Growth and drug sensitivity screening of gene deletion mutants, related to Figure 4.

A. Growth and drug sensitivity of gene deletion mutants. Cells were cultivated in EMM+S medium for 48 h.

B. Growth of gene deletion mutants. Cells were inoculated on EMM+E or EMM+S medium for five days.

C. Growth of *fsf1*Δ, *fkh1*Δ, and double deletion (*fsf1*Δ *fkh1*Δ) cells. Data represent the mean ± SD (n = 4).

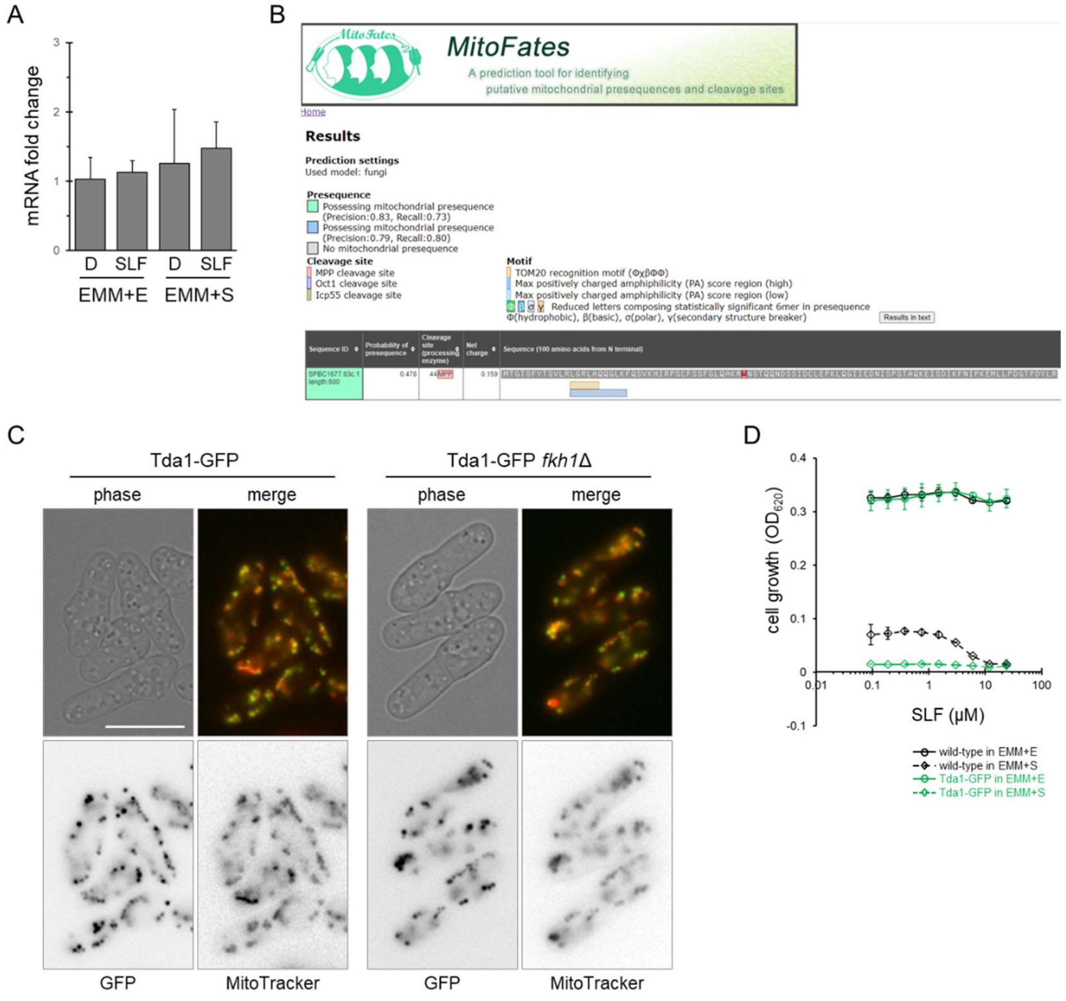

Figure S8. mRNA expression and protein localization of Tda1, related to Figure 6.

A. mRNA level of *tda1*. Cells were cultivated in EMM+E or EMM+S medium, then exposed to DMSO (D) or SLF for 2 h. Data represent the mean ± SD (n = 3).

B. The putative mitochondrial targeting sequence (MTS) of Tda1, deduced by MitoFates (Fukasawa et al., 2015).

C. Cellular localization of Tda1-GFP. Cells were cultivated in EMM+E medium. Mitochondria were stained by MitoTracker Red CMXRos. Merged images are shown in upper right panels.

D. Growth and drug sensitivity of cells expressing Tda1-GFP. Wild-type or cells expressing Tda1-GFP were cultivated in EMM+E or EMM+S medium. Data represent the mean ± SD (n = 3).

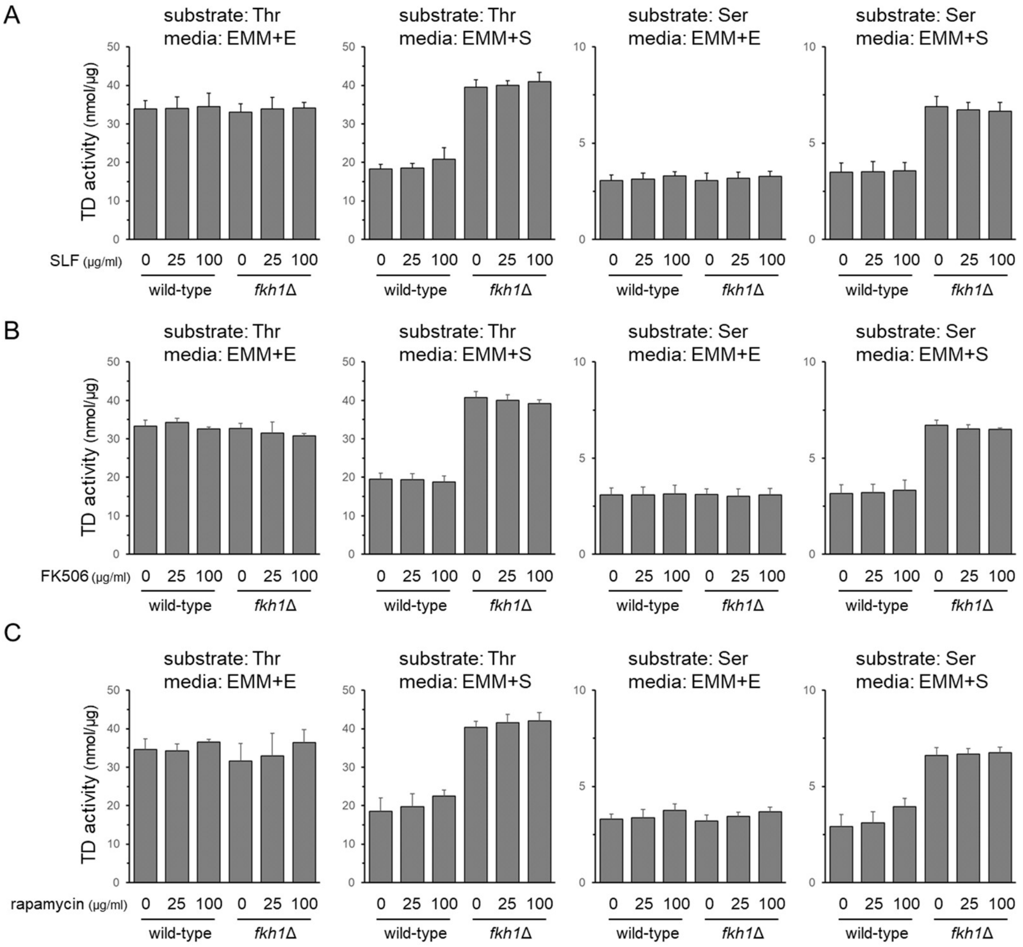

Figure S9. Effect of FKBP12 inhibitors on the cellular TD activity, related to Figure 6. TD activity was measured in the presence of FKBP12i: SLF (A), FK506 (B), or rapamycin (C). Substrates are threonine (10 mM) or serine (20 mM), and the reaction time was 10 minutes. Data represent the mean ± SD (n = 3).

Table S1. List of 322 metabolites with their relative abundance, related to Figure 3

Table S2. List of 110 metabolites with their absolute quantity, related to Figure 3

Table S3. List of genes involved in serine biosynthesis and catabolism, related to Figure 4*

systematic ID	gene name	gene product**	gene deletion viability**
SPAC24C9.12c	shm1	serine hydroxymethyltransferase Shm1 (predicted)	viable
SPAC18G6.04c	shm2	serine hydroxymethyltransferase Shm2 (predicted)	viable
SPAC1F12.07	ser1	phosphoserine aminotransferase (predicted)	viable
SPBC3H7.07c	ser2	phosphoserine phosphatase Ser2 (predicted)	viable
SPCC364.07	ser3	D-3 phosphoglycerate dehydrogenase Ser3 (predicted)	viable
SPCC320.14	sry1	serine racemase Sry	viable
SPAC1039.06		D-serine ammonia-lyase activity (predicted)	viable
SPAC17G6.15c	fsf1	mitochondrial carrier, serine Fsf1 (predicted)	viable
SPBC1685.07c	avt5	vacuolar amino acid transmembrane transporter Avt5	viable
SPBC1677.03c	tda1	threonine ammonia-lyase Tda1	inviable
SPAC19A8.15	trp2	tryptophan synthase (predicted)	inviable
SPCC1442.12	pps1	CDP-diacylglycerol-serine O-phosphatidyltransferase Pps1	depends on conditions

*Metabolic pathways for the gene products are shown in Figure S3D.

**Gene product and viability are based on PomBase (https://www.pombase.org/).

Table S4. Yeast strains used in this study, related to STAR Methods

strain	genotype	comment
JY1	*h⁻*	lab stock
NI1145	*h⁻ fkh1::natR*	Ikai et al. 2011.*
MS003-4	*h⁻ fkh1::kanR leu1-32*	this study
MS086-88	*h⁺ fkh1::natR*	this study
MS013-15	*h⁻ fkh1::kanR leu1⁺ << Pnmt1-FFH*	this study
MS016-18	*h⁻ fkh1::kanR leu1⁺ << Pnmt1-fkh1-FFH*	this study
MS019-21	*h⁻ fkh1::kanR leu1 ⁺ << Pnmt1-YFH*	this study
MS022-24	*h⁻ fkh1::kanR leu1 ⁺ << Pnmt1-fkh1-YFH*	this study
MS063-64	*shm1::kanR*	this study
MS067	*h⁻ sry1::kanR*	this study
MS068	*sry1::kanR*	this study
MS069-70	*h⁻ fsf1::kanR*	this study
MS073	*SPAC1039.06::kanR*	this study
MS074	*h⁻ SPAC1039.06::kanR*	this study
MS075-76	*h⁻ ser3::kanR*	this study
MS079	*h⁻ fkh1::kanR*	this study
MS080	*h⁺ fkh1::kanR*	this study
MS084	*h⁻ ser3::kanR*	this study
MS085	*h⁻ fsf1::kanR*	this study
MS089-90	*shm2::kanR*	this study
MS094-96	*fkh1::natR fsf1::kanR*	this study
MS100-102	*h⁻ ser1::kanR*	this study
MS103-105	*h⁻ ser2::kanR*	this study
MS106-108	*h⁻ avt5::kanR*	this study
MS139-141	*h⁻ tda1::kanR-Pnmt1-tda1*	this study
MS142-144	*h⁻ tda1::kanR-Pnmt41-tda1*	this study
MS145-147	*h⁻ tda1::kanR-Pnmt81-tda1*	this study
MS148,150	*h⁻ tda1::kanR-Pnmt1-tda1 fkh1::natR*	this study
MS149	*h^{90} tda1::kanR-Pnmt1-tda1 fkh1::natR*	this study
MS151-152	*h⁻ tda1::kanR-Pnmt41-tda1 fkh1::natR*	this study
MS153	*h⁺ tda1::kanR-Pnmt41-tda1 fkh1::natR*	this study
MS157-159	*h- tda1::tda1-GFP-kanr fkh1::natR*	this study
MS160-162	*h⁻ tda1::tda1-GFP-kanR fkh1::natR*	this study
MS169, 171	*tda1::kanR-Pnmt81-tda1 fkh1::natR*	this study

*Ikai N, Nakazawa N, Hayashi T, Yanagida M. (2011) The reverse, but coordinated, roles of Tor2 (TORC1) and Tor1 (TORC2) kinases for growth, cell cycle and separase-mediated mitosis in *Schizosaccharomyces pombe*. Open Biol. 1, 110007.

Table S5. Oligo DNAs used in this study, related to STAR Methods

Name	Sequence	Purpose
fkh1-DF1	CTTCAAACCAGCTACATAGCTC	gene deletion
fkh1-DF2	TTAATTAACCCGGGGATCCG-GGTTAATGAGCAAACCCGAAAG	gene deletion
fkh1-DR1	ACTCTAGCAAGTGCCGTATATG	gene deletion
fkh1-DR2	GTTTAAACGAGCTCGAATTC-GCATCTTACTAGCGGTTGTTAC	gene deletion
fkh1-DC1	GTGCTGCTCATTCTATTCCATC	gene deletion
fkh1-DC2	TTCTGAGAACAAGGCAATGG	gene deletion
ser1-DF1	GCTGAGACAGCCTTTGGACT	gene deletion
ser1-DF2	TTAATTAACCCGGGGATCCG-CCGTGAGCTCAAGCAGAAGT	gene deletion
ser1-DR1	TTGTGGGCACAACAGAAAGC	gene deletion
ser1-DR2	GTTTAAACGAGCTCGAATTC-GATGTGGGAGCTGTTGGTCC	gene deletion
ser1-DC1	ACCGACAGGATGCAATACGC	gene deletion
ser1-DC2	ACGTCTGGCCCAATCGATTG	gene deletion
ser2-DF1	AGACACCAAAAGCAGTAGCGG	gene deletion
ser2-DF2	TTAATTAACCCGGGGATCCG-GCGTTGTTTTCGGCAAGTCC	gene deletion
ser2-DR1	CCCGTGCACAAAATCGATGC	gene deletion
ser2-DR2	GTTTAAACGAGCTCGAATTC-GGCGCTGTCAAGAACTCTGA	gene deletion
ser2-new-DC1	ACATGAAGATCAAGTCCGAATTCC	gene deletion
ser2-new-DC2	TGGGACTACATTGGTGAGACC	gene deletion
avt5-DF1	CTGCTGGCAAACCAGAGTAGAG	gene deletion
avt5-DF2	TTAATTAACCCGGGGATCCGAGCGTATGAGTATGCAAACTGATCG	gene deletion
avt5-DR1	ACCTGAAGCTGCTGCATATCC	gene deletion
avt5-DR2	GTTTAAACGAGCTCGAATTCTGCGTTTAGCACTTCGCTCG	gene deletion
avt5-DC1	TCCTTGTGATCAACGTTTGACAGTG	gene deletion
avt5-DC2	TCTGTGAGATCGGGAACTGTACTG	gene deletion
tda1-nmtprm-NF1	TGGATCTACTTAACAGCTTTGCC	promoter exchange
tda1-nmtprm-NF2	GTTTAAACGAGCTCGAATTC-TGAATTGATCCCTTCTGGATTAAGC	promoter exchange
tda1-nmtprm-NR1	ACTGGAACACCGGTACTTTCAG	promoter exchange
tda1-nmtprm-NR2	TATAGTCGCTTTGTTAAATCATG-ACTGGAACGAGTTTTTACACTTCG	promoter exchange
tda1_nmtprm_NC1	ATAGCTTTGAAATTCTCTCAGCTTG	promoter exchange
tda1_nmtprm_NC2	AGCAATGACTCCATTTTTCAATGAC	promoter exchange
tda1_GFP_CF1	TGAGTGTCACTATTCCTGAGCG	C-terminal GFP tag
tda1_GFP_CF2	TTAATTAACCCGGGGATCCG-TTTACGAAGATAGCGCAAGTAAACG	C-terminal GFP tag
tda1-DR1	AAGCACAGGATGCTTTAAGGTC	C-terminal GFP tag
tda1-DR2	GTTTAAACGAGCTCGAATTC-TCGCCATCTTTGAATCCTATAGACG	C-terminal GFP tag
tda1_GFP_CC1_0405	TGGTATGAAGCGTTATGTCGC	C-terminal GFP tag
tda1-DC2	AACCAGAAGCTTATGTTGATTTGG	C-terminal GFP tag
qPCR-actin-F	ACTACCGCCGAACGTGAAAT	qPCR
qPCR-actin-R	TACCAGGTCCGCTCTCATCA	qPCR
tda1_qPCR_F	TCTGAAAGTACCGGTGTTCCAG	qPCR
tda1_qPCR_R	AGCGGAACAAGCAATGACTC	qPCR
